# Applications of Prodigiosin Extracted from Marine Red Pigmented Bacteria *Zooshikella* sp. and Actinomycete *Streptomyces* sp.

**DOI:** 10.3390/microorganisms8040556

**Published:** 2020-04-13

**Authors:** Chatragadda Ramesh, Nambali Valsalan Vinithkumar, Ramalingam Kirubagaran, Chidambaram Kulandaisamy Venil, Laurent Dufossé

**Affiliations:** 1 National Institute of Oceanography (CSIR-NIO), Dona Paula 403004, India; 2 Atal Centre for Ocean Science and Technology, ESSO-NIOT, Dollygunj, Port Blair, Andaman and Nicobar Islands 744103, India; vinith@niot.res.in; 3 Marine Biotechnology Group, ESSO-National Institute of Ocean Technology (NIOT), Ministry of Earth Sciences (Govt. of India), Chennai 600100, India; kirubagar@gmail.com; 4 Department of Biotechnology, Anna University, Coimbatore 641046, India; ckvenil@gmail.com; 5 Chemistry and Biotechnology of Natural Products, CHEMBIOPRO, Université de La Réunion, ESIROI Agroalimentaire, 15 Avenue René Cassin, F-97744 Saint-Denis CEDEX 9, France

**Keywords:** *Zooshikella* sp., *Streptomyces* sp., marine sediment, antibacterial activity, prodigiosin

## Abstract

This study is aimed to determine the distribution, diversity and bioprospecting aspects of marine pigmented bacteria (MPB) isolated from pristine Andaman Islands, India. A total of 180 samples including seawater, sediment, marine plants, invertebrates, and vertebrates were collected and investigated for isolating pigmented bacteria. Results revealed that sediment, invertebrates, and seawater samples were colonized with a greater number of pigmented bacteria pertains to 27.9 × 10^3^ CFU/mL, 24.1 × 10^3^ CFU/mL and 6.7 × 10^3^ CFU/mL respectively. Orange (21.6 × 10^3^ CFU/mL) and red (8.0 × 10^3^ CFU/mL) MPB were predominant than other pigmented bacteria. Fourteen potential MPB were selected based on their intense pigmentation and tested for bioactive nature and food colorant applications. Out of 14, two red pigmented strains BSE6.1 & S2.1 displayed potential multifaceted applications, such as antibacterial, antioxidant, food colorant, and staining properties. Brown pigmented strains CO8 and yellow pigmented strain SQ2.3 have displayed staining properties. Chemical characterization of red pigment using TLC, HP-LC, GC-MS, FT-IR and ^1^H-NMR analysis revealed prodigiosin as a main chemical constituent. Pure form of prodigiosin compound fractions obtained from both the strains displayed effective antibacterial activity against different human pathogens. MIC and MBC assays revealed that S2.1 requires 300 µg and 150 µg, respectively, and BSE6.1 require 400 µg concentrations of pigment compound for complete inhibition of *S. aureus* subsp. *aureus*. On the basis of 16S rRNA sequence analysis, strains S2.1 and BSE6.1 were identified as *Zooshikella* sp. and *Streptomyces* sp. and assigned under the GenBank accession numbers: MK680108 and MK951781 respectively.

## 1. Introduction

Marine bacterial communities are known to play an important role in regulation of ecological and biogeochemical processes and shaping the biosphere [1]. Some microorganism has enormous potentiality to produce diverse striking traits such as production of pigments and biomolecules etc. Pigment production in some microbes is regulated via the quorum sensing mechanism [2]. Several marine pigmented bacterial species have demonstrated various biological activities such as antimicrobial, anticancer and immunosuppressive activities [3]. Recent studies on natural products and microbial autecology science have increased requisite demand on investigating novel and alternative resources for eco-friendly and aesthetic natural products like bacterial pigments for different biomedical and industrial applications.

Unlike pigmentation in terrestrial bacteria, marine pigmented bacteria display a wide array of pigment hues including black, brown, golden, indigo, silver, red, yellow, blue, violet, green, orange and fluorescent green [4,5]. In the past five decades, over more than 10,000 marine microbial metabolites have been characterized [6]. However, pigment molecules from marine bacteria are poorly understood and extensive studies are yet to be focused on this aspect. In recent research, microbial pigments are emphasized as unending and untapped natural sources for the applications of food grade pigments, antimicrobial, antidiabetic, anticancer and antiulcer agents for pharmaceutical industries [7]. Currently, scientists and food industries are seeking for such natural pigments from marine bacteria due to their biological functional attributes. These molecules are not fully explored from marine microbes and are still untouched when compared to microbes of terrestrial origin.

Several marine Gram-positive and Gram-negative bacteria produce pigments on usual culture media. The characteristics of these bacteria resemble members of the genera *Alteromonas*, *Corynebacterium*, *Flavobacterium*, and *Pseudomonas* [4]. The awareness of natural products and microbial autecology science has increased requisite demand on investigating newer and alternative resources for eco-friendly natural products like bacterial pigments for different biomedical and industrial applications. Since the use of synthetic pigments and their by-products in various applications such as cosmetics, food and pharmaceuticals appear to show significant impact on human health as toxic, carcinogenic and teratogenic properties [4], several studies have been undertaken to investigate natural pigment sources from plants and terrestrial bacteria and fungi [8]. Therefore, the present study has focused to investigate the potential applications of various pigment molecules producing bacteria from different marine biota.

Marine bacterial natural characters such as pigments, medicinal properties, and nutrients production like vitamins are dependent on season and geographical conditions. Andaman and Nicobar Islands are located about 1200 km far from mainland consisting a chain of 836 Islands from North to Southern on longitude 93°–94° East and latitude 6°–17° North. Out of the 836 islands, 38 islands are inhabited, and eight islands are covered under various settlement programmes. These islands have a rich marine biodiversity and erratic weather conditions, which make these islands different from other geographic places. Studies on marine bacterial diversity are a key phenomenon in understanding their biogeography, community assembly and ecological role. In this context, very little is known about pigmented bacterial distribution in marine environment. Studies carried out on pigmented bacteria are very limited in India, and so far, particularly in Andaman studies are scarce [9]. This necessitated the present study to explore pigmented bacterial for food and drug applications.

## 2. Material and Methods

### 2.1. Study Area

**Figure 1:**
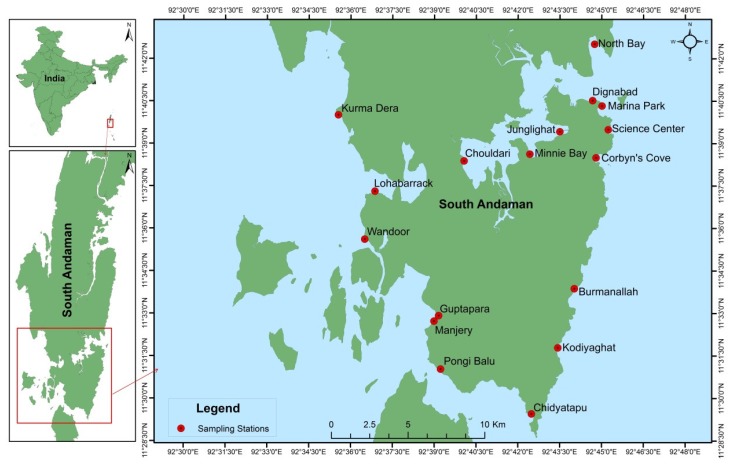
Sampling stations in South Andaman Island.

Sampling was conducted at seventeen different stations located around the coastal regions of South Andaman Island during low tide. The GPS coordinates (Latitude and longitude) of the seventeen stations are as follows: Corbyn’s Cove (11°38′29.78″ N & 92°44′47.99″ E), Burmanallah (11°33′52.24″ N & 92°44′01.51″ E), Kodiyaghat (11°31′47.16″ N & 92°43′25.97″ E), Marina Park (11°40′19.76″ N & 92°45′00.84″ E), Mini Bay (11°38′37.46″ N & 92°42′25.53″ E), Dignabad (11°40′31.16″ N & 92°44′40.68″ E), Science Center (11°39′29.32″ N & 92°45′14.40″ E), Wandoor (11°35′36.79″ N & 92°36′30.25″ E), Lohabarrack (11°37′18.59″ N & 92°36′52.18″ E), Pongi Balu (11°31′01.86″ N & 92°39′13.74″ E), Chidiyatapu (11°29′27.24″ N & 92°42′29.38″ E), Kurma Dera (11°40′00.56″ N & 92°35′33.24″ E), Chouldari (11°38′22.97″ N & 92°40′03.93″ E), Manjery (11°32′43.40″ N & 92°38′59.39″ E), Guptapara (11°32′55.12″ N & 92°39′09.40″ E), Junglighat (11°39′25.09″ N & 92°43′30.07″ E) and North Bay (11°42′30.73″ N & 92°44′44.81″ E) (Figure 1).

### 2.2. Collection of Samples

Samples were categorized into two major groups such as biotic and abiotic samples. Biotic samples are comprised of floral and faunal components (Supplementary Table S1). All samples were collected following standard guidelines specified in previous studies [9]. Floral samples including seaweeds, seagrass and mangrove roots were collected using hand pick method following aseptic conditions. Faunal components spanning lower order group of invertebrates to higher order group of vertebrates. Several swab samples were collected from sessile, fragile, rare and dead samples in the field using sterile cotton swabs procured from HiMedia, Mumbai.

Abiotic samples comprised of various materials were as follows: seawater, sediment and Muscovite (common mica). Sea water samples were collected in a sterile water sampler bottle. Sediment samples were collected with a sterile hand corer and transferred it into a sterile sample container. A total of 180 samples including biotic and abiotic samples were collected for investigating the pigmented bacterial presence (Supplementary Table S1). All the samples were collected only once and tested for the occurrence of pigmented bacteria. Samples were placed inside an ice-box in cooled conditions at 4–8°C in polystyrene container and transported to laboratory within one hour of sampling for further bacteriological studies.

### 2.3. Cultivation and Isolation of Pigmented Bacteria

Different media such as Marine agar, Nutrient agar, Luminescent agar, Photobacterium agar, Pseudomonas fluorescence agar, Pseudomonas pyocyanin agar, Bacillus agar, Seawater complete agar, Casitose agar, were employed to isolate different pigmented bacteria. All media were prepared using Millipore water and autoclaved in EQUITRON Autoclave (#7440FA) at 121 °C for 15 min. Swab samples obtained from different floral and faunal samples were directly swabbed onto different media plates. Sediment and mica sample aliquots were prepared by diluting 1 gm of each sample in separate 9 mL of sterile seawater (w/v) and used for bacteriological analysis [10]. A 100 µL of seawater and sediment aliquots were processed using spread plate method [11,12]. Simultaneously, these plates were incubated in bacteriological incubator at 32 °C and at room temperature for 7 to 10 days. After incubation period, colonies showing pigmentation were picked with sterile toothpicks and sub-cultured by streaking method to obtain pure single isolated colonies.

### 2.4. Storage

Pure single colony pigmented isolates of 14 strains were maintained on agar plates or agar slants at 4 °C or at room temperature up to 2–3 months. Glycerol stocks were maintained at −20 °C in 5 mL round bottom polypropylene Cryo Vials (Abdos^TM^) for longer period and were prepared by inoculating 1.5 mL of sterile glycerol (30%) to 3.5 mL of strain specific broth for future studies.

### 2.5. Molecular Characterization of Pigmented Bacteria

#### 2.5.1. Genomic DNA Extraction

Genomic DNA’s of selected pigmented bacterial isolates were extracted using QIAamp DNA Blood Mini Kit, procured from QIAGEN. Initially, lysis of Gram-negative and Gram-positive bacterial cells were achieved by mechanical shearing by sonication for 5 min and bead homogenizer (FastPrep-24^TM^ 5G benchtop homogenizer, MP Biomedicals), followed by enzymatic lysis with proteinase-K (20 mg/mL) and lysozyme (10 mg/mL) respectively for 30 min. Further steps are performed as per the Quick-Start Protocol guidelines of QIAGEN. The isolated DNA was quantified using DeNovix DS-11 + Spectrophotometer by measuring absorptions. Only high-quality DNA with A_260_/A_280_ ratio of 1.8 to 2.0 were used. Intense pigment producing bacterial strains were subjected to 16S rRNA sequence analysis for identification. Further, two potential pigmented bacterial strains are characterized by a combination of molecular techniques such as RAPD & RFLP, in order to determine the degree of inter- and intraspecific variability for future studies.

#### 2.5.2. PCR Amplification of 16S rRNA Analysis

PCR amplification of 16S rRNA was carried out in Applied Biosystems Version 2.0 using the bacterial consensus primers 27F [5’-AGA GTT TGA TCC TGG CTC AG-3’] and 1492R [5’-GGT TAC CTT GTT ACG ACT T-3’] universal forward and reverse 16S rRNA primers [13]. The PCR of the genomic DNA isolate were conducted in a final volume of 25 μL. The reaction mixture contained 10× PCR buffer, 25 mM MgCl_2_, 10 μM DNTP’s, 1 U of Taq DNA polymerase, 10 pmol of each forward and reverse oligonucleotide primers and approximately 20 ng of genomic DNA. The amplification profile consisted of an initial denaturation at 94 °C for 3 min, followed by 35 cycles at 94 °C for 30 sonds, 52 °C for 1 min and 72 °C for 1 min. This was followed by a final extension step of 72 °C for 5 min. The samples were held at 4 °C until further sequence analysis.

#### 2.5.3. RAPD-PCR Analysis

Two red pigment strains BSE6.1 and S2.1 were subjected to RAPD analysis with twenty different primers, which are procured from Eurofins (Bangalore, India). Chromosomal DNA of strains BSE6 & S2 have been used to generate the discrimination profile for identifying these strains rapidly using this analysis. The twenty primers used for RAPD analysis were as follows: OPA-07 GAAACGGGTG, OPA-12 TCGGCGATAG, OPB-01 GTTTCGCTCC, OPB-15 GGAGGGTGTT, OPC-05 GATGACCGCC, OPD-10 GGTCTACACC, OPE-11 GAGTCTCAGG, OPF-05 CCGAATTCCC, OPJ-01 CCCGGCATAA, OPJ-12 GTCCCGTGGT, OPR-05 GACCTAGTGG, OPR-12 ACAGGTGCGT, OPU-06 ACCTTTGCGG, OPU-13 GGCTGGTTCC, OPU-17 ACCTGGGGAG, OPV-06 ACGCCCAGGT, OPW-10 TCGCATCCCT, OPX-08 CAGGGGTGGA, OPX-13 ACGGGAGCAA, and OPZ-19 GTGCGAGCAA.

The final reaction volume of 25 µL was used to perform RAPD-PCR amplification, following the optimized conditions of: 94 °C for 5 min, 35 cycles of 94 °C for 30 s, 35 °C for 30 s, 72 °C for 3 min, and final extension at 72 °C for 7 min. Then PCR products were resolved in 1.2% TAE agarose gels at 80V for 40 min in electrophoresis unit. The molecular markers of 100 bp and 1 Kb DNA ladders were used as a size markers. Subsequently, the different polymorphic DNA banding patterns observed were recorded.

#### 2.5.4. RFLP Analysis of Amplified 16S rRNA

Four different restriction enzymes such as *AluI* (BioLabs, New England), *HindIII*, *BamHI* and *EcoRI* (HiMedia, Mumbai, IndiaThe crude products thus obtained were) were used to generate species specific ARDRA banding patterns. Approximately, 7 µL of PCR amplified 16S rRNA products of BSE6.1 and S2.1 were digested with 1 U of each restriction enzyme following manufactures guidelines (HiMedia, Mumbai). The reaction mixture in a total volume of 25 µL containing 7 µL of amplified DNA, 2 µL of restriction enzyme, 5 µL of 10× reaction buffer and 11 µL of molecular grade water (HiMeida). The reaction mixtures were incubated at 37 °C for 15 to 30 min or left overnight, followed by inactivation of enzymes by heating at 65 °C for 15 min. Then, the reaction products were analysed by 3% (*w*/*v*) agarose gel electrophoresis in TAE buffer containing 3 µL of ethidium bromide in 30 mL gel.

#### 2.5.5. Sequencing and Phylogenetic Analysis

All the isolates were sequenced with an automatic sequencer Applied bio system, Foster City, USA. Raw sequences alignment and quality were checked with MEGA 6 software [14]. The Black box chimera check (B2C2) software was used to check chimera formations [15]. The partial 16S rRNA gene sequences of these isolated strains were compared with those in the public databases including GenBank, DDBJ, EMBL, ENA, EzBioCloud, LeBibi database, Ribosomal Database (RDP), CAMERA 2.0 Portal and EzTaxon-e database to search for the nearest phylogenetic neighbour. Species level identification was determined by the 16S rRNA sequence similarity of 99% with that of the prototype sequence available in GenBank. Sequence alignment and comparison was performed using the multiple sequence alignment program ClustalW [16]. Sequence similarities and nucleotide gaps were also calculated and edited using BIOEDIT software. Neighbour-joining method was chosen to construct and to calculate the evolutionary distance of the phylogenetic tree using MEGA6 software [17]. Bootstrap analysis was performed by applying 1000 resamplings to evaluate the topology of neighbour joining tree [18].

#### 2.5.6. Nucleotide Sequence Accession Numbers

Nucleotide sequences of 16S rRNA gene were deposited in the NCBI GenBank under assigned accession numbers.

### 2.6. Extraction and Preparation of Crude Pigmented Compounds

Pure pigmented bacterial strains were cultured in 100 mL of respective broth as mentioned above for 5–7 days under shaking with 200 rpm (ORBITEK-Scigenics Biotech) at 28 °C in 250 mL Erlenmeyer flasks [9,19]. This was followed by centrifugation at 10,000 rpm for 10 min (SIGMA 3–30KS). The raffinate and cell pellets of pigmented bacterial strains were extracted with an equal volume of methanol until the pellet was colourless. The organic phases were collected using separating funnel and transferred into separate glass bottles for rotary evaporation. Solvent from the extracts was removed under reduced pressure at 40 °C by rotatory evaporation (BÜCHI Rotavapor R-205, BÜCHI Vacuum controller V-800 & BÜCHI Chiller B-740). The crude products thus obtained were redissolved in 1 mL of the same solvent for further applications.

### 2.7. Antibacterial Properties of Pigment Extracts

#### 2.7.1. Human Bacterial Pathogens

Human bacterial pathogenic culture in lyophilized powder form was purchased from Microbial Type Culture Collection and Gene Bank (MTCC), CSIR-Institute of Microbial Technology, Chandigarh, India. The *Staphylococcus aureus* subsp. *aureus* MTCC1430 was obtained and retrieved on specified media given in the guidelines of MTCC. This pathogen was chosen due to its pathogenicity, causing a range of minor skin infections to life-threatening diseases.

#### 2.7.2. Preparation of Human Pathogenic Bacterial Inoculums

Stringent aseptic conditions were adopted for preparation of aforementioned pathogenic bacterial inoculums. A loop full of each human pathogenic bacterial culture was inoculated into separate 5 mL glass tubes containing enriched broth medium, i.e., Muller Hinton broth. These inoculated culture tubes were incubated at 35 °C for overnight. Broth cultures with an optical density of OD_600_ = 1 were used as inoculum suspensions for antibacterial assay.

#### 2.7.3. Antibacterial Assays

Antibacterial activity assay was performed according to Kirby-Bauer well diffusion and disc diffusion method [20]. Using sterile cotton swabs, overnight grown inoculum suspensions of pathogenic bacteria that gives a lawn density of approximately 10^5^ to 10^8^ CFU/mL were swabbed onto the surface of Mueller-Hinton agar plates. Lids of petri dishes were left ajar for 2 to 5 min to remove excess surface moisture. Wells were made on media plates using cork borers and impregnated with 100 µL of crude extracts. Sterile disks treated with equal volume of respective solvents of each extract were used as negative control. After complete diffusion of extractions into media, plates were incubated at 35 °C for 24 h. The inhibition zones around the disc were measured including disc diameter (6 mm).

#### 2.7.4. Minimum Inhibitory Concentration (MIC) and Minimum Bactericidal Concentration (MBC) Assays

MIC and MBC were defined as the lowest concentration of pigment compounds which inhibited the visible growth of a microorganism after overnight incubation with specific concentration of pigment compounds. Tube dilution method was carried out using aseptic conditions as per the standard protocols [21,22]. Sterile test tubes were numbered 1 to 7. The dried pigment compounds of BSE6.1 and S2.1 with concentrations of 400 µg/mL and 300 µg/mL respectively were diluted in first test tube containing 2 mL of Muller Hinton broth (MHB). And, to all other test tubes 1 mL of MHB was added. From the 1st test tube 1 mL was transferred to the sond tube and the same was followed till fifth tube (concentrations are given in the figures). From fifth test tube 1 mL was discarded. To all these tubes 100 µL of multiple infections causing pathogen *S. aureus* subsp. *aureus* MTCC1430 culture with OD value 1 was inoculated and vortexed gently. Sixth tube containing MHB serves as negative control and seventh tube with *S. aureus* inoculum in MHB serves as positive control. All these tubes were incubated at 35 °C for 24 h. The lowest concentration that inhibited the growth of *S. aureus* completely was defined as MIC. MBC was measured by transferring 100 µL of culture suspensions from each tube used in MIC assay onto Muller Hinton agar and incubated at 35 °C for 24 h or longer. The plates that have not formed any growth was considered as MBC.

### 2.8. Food Colorant and Staining Applications of Pigments

Crude concentrated pigment extracts obtained from potential pigmented strains were investigated for food colorant and staining applications. For food colorant application, agar jellies were prepared by dissolving 5% agar in 100 mL distilled water, heated and melted until agar residues disappear. At 45 °C, different pigment extracts with concentration of 500 µg were dissolved separately in melted agar solution and poured into jelly making plate. After complete solidification, jellies were removed and kept at 4 °C for few months to see the long lasting capability of pigment.

For staining application, *Tridax procumbens* stem was used for testing the staining capacity of different pigment extracts. Transverse stion of *T. procumbens* stem were dipped for two min and observed under microscope to see any cell specific (epidermis, xylem, phloem, parenchyma) staining is occurred.

### 2.9. Statistical Analysis

All experiments were performed in triplicates for error analysis and the values derived from triplicates were expressed as means ± standard error and presented in each graph.

## 3. Characterization of the Pigment

### 3.1. Thin Layer Chromatography Analysis (TLC)

Compound characterization was only performed to red pigment strains BSE6.1 and S2.1 due to their effective antimicrobial activities. TLC was performed on silica gel coated aluminium sheets (60 F254, Merck) procured from Merck. The crude extract was loaded on the line drawn on silica-coated plates using a fine capillary tube (2–5 µL) and immersed in the solvent system just below the sample loaded line. Different solvent systems were tested to obtain the best separation of compounds. The best optimized solvent system used for separating red pigment compound of strain BSE6.1 and S2.1 were methanol, hexane and dichloromethane at the ratio of 1:3:6. The plates were removed after the solvent reaches the 3/4th of the plate, dried, and examined to count the number of distinct pigment bands separated under normal light, and fluorescent spots in a UV-chamber at 366 nm. Subsequently, R*f* (retention factor) values were calculated using the below formula and compared with the R*f* values reported in previous studies. The TLC-pure compounds of BSE6.1 and S2.1 were further subjected to HPLC, UV-Visible spectroscopy, infrared spectroscopy (FT-IR), GC-MS and proton NMR. Analytical part of these samples using HPLC, FT-IR, GC-MS and NMR were carried out at Indian Institute of Chemical Technology, Hyderabad.

(1) $$Rf=\frac{\mathrm{Distance}\;\mathrm{travelled}\;\mathrm{by}\;\mathrm{the}\;\mathrm{compound}}{\mathrm{Distance}\;\mathrm{travelled}\;\mathrm{by}\;\mathrm{the}\;\mathrm{solvent}\;\mathrm{front}}$$

### 3.2. High Performance Liquid Chromatography Analysis (HPLC)

HPLC has emerged as the most versatile and robust technique for isolation and identification of metabolites like pigments. Distinct bands observed in TLC were separated by scrapping with fine pointed surgical blade and dissolved in same solvent and vortexed well until the silica gel becomes colourless. The pigmented solvent was separated carefully from silica gel and centrifuged at 10,000 rpm for 7 min and pipetted out into a new tube and used for HPLC.

### 3.3. UV-Visible Spectrophotometry

Purified red pigment compounds of strains BSE6.1 and S2.1 were dissolved in methanol and absorbance was scanned using spectrophotometer (PerkinElmer UV/VIS spectrophotometer Lambda25) between 200 and 750 nm to obtain the maximum absorption wavelength associated with these pigments. Prior to scanning, instrument was calibrated with ethanol as blank.

### 3.4. Fourier-Transform Infrared Spectroscopy (FT-IR)

Infrared spectra of red pigments in ethanol solvents were collected by placing the pigments samples on separate KBr pellets. Absorption spectra were collected in the range of 500 to 4000 cm^−1^ using Thermo Nicolet Nexus 670 Spectrometer with DTGS-KB detector and resolution 4 cm^−1^. The IR spectra were identified by using FT-IR spectra library [23].

### 3.5. Gas Chromatography and Mass Spectrometry (GC-MS) Analysis

GC-MS was performed to identify the major and minor compounds present in purified red pigments. The following samples BSE6.1 and S2.1 were analysed by GC-MS technique on Agilent6890 Series GC-MS, using HP-5MS 30 m × 0.25 mm × 0.25 µm column with 1.2 mL flow rate. Different compounds detected in the spectra were identified using NIST05a.L library.

### 3.6. Proton NMR Spectroscopy Analysis

Proton (^1^H) NMR spectroscopy was used to detect the magnetic fields around the atomic nuclei. The proton spectral data of red pigments of BSE6.1 and S2.1 were generated in deuterated chloroform (CDCl_3_) solution at 400 MHz, using a Bruker Avance-400 solid NMR spectrometer.

## 4. Results and Discussion

### 4.1. Isolation of Marine Pigmented Bacteria

**Figure 2:**
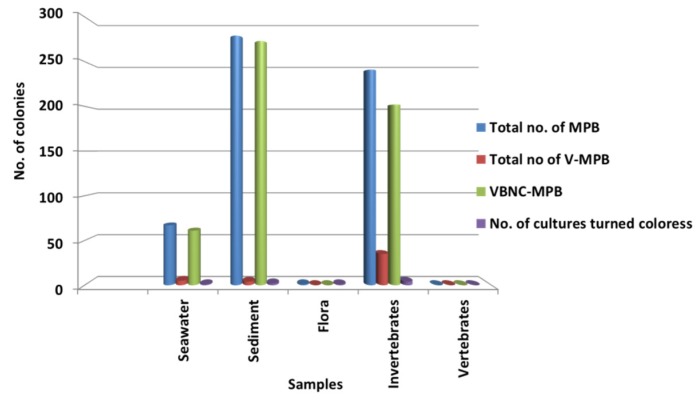
Dominance of pigmented bacteria in different sample groups.

**Figure 3:**
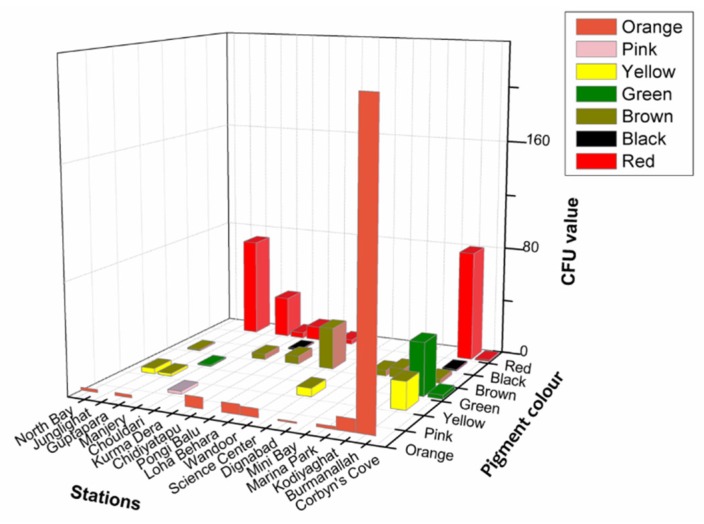
3D plot showing abundance of type of pigmented bacteria from different stations.

**Figure 4:**
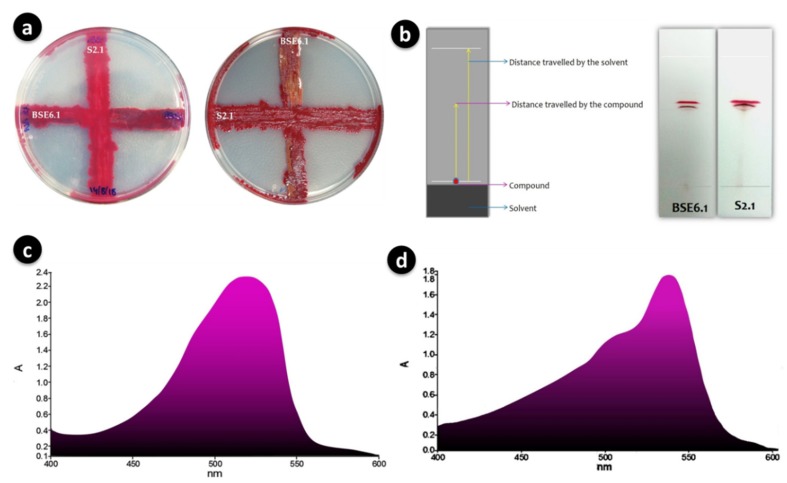
Cross streaking of strains BSE6.1 and S2.1 showed no positive antagonistic activity (**a**); Separation of main red pigment fractions of BSE6.1 & S2.1 by TLC (**b**); Maximum absorption wavelengths of 528 nm for strain BSE6 (**c**) and 538 for strain S2.1 (**d**).

**Figure 5:**
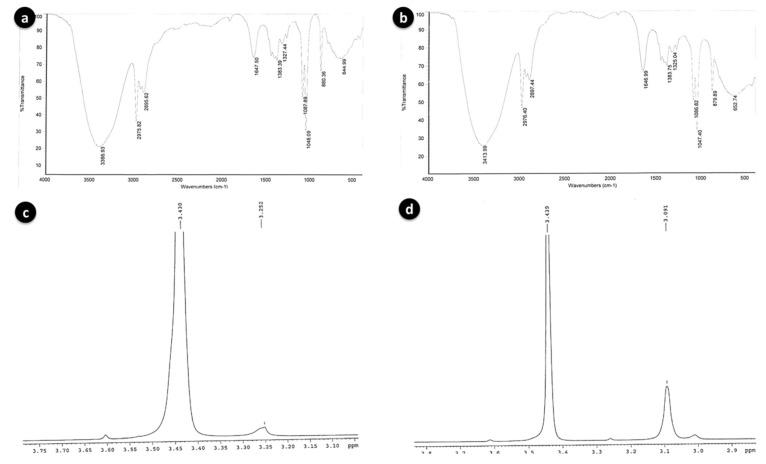
FT-IR spectra of BSE6.1 (**a**) and S2.1 (**b**); Proton NMR spectra of BSE6.1 (**c**) and S2.1 (**d**).

Significantly in all the examined samples, pigmented bacteria were more abundant in sediment and invertebrate samples (Figure 2). Although different pigmented isolates including yellow, orange, black, greenish, brown and red were isolated (Figure 3, Supplementary Material, Figures S1–S3), more frequently and abundantly orange, red, brown and green were observed in this study (Figure 4 and Figure 5). Among all the sampling stations, Burmanallah revealed the potential source of pigmented bacterial diversity (Figure 3). Totally, viable pigmented bacteria account for 1.4 to 14%, viable but non-culturable marine pigmented bacteria (VBNC-MPB) for 90 to 97%, pigmentation lost cultures with 0.7%–1.6% and diffusible pigment producing bacteria are 1%. Most of the pigmented bacterial isolates have grown on agar media after 10–20 days of incubation.

**Figure 6:**
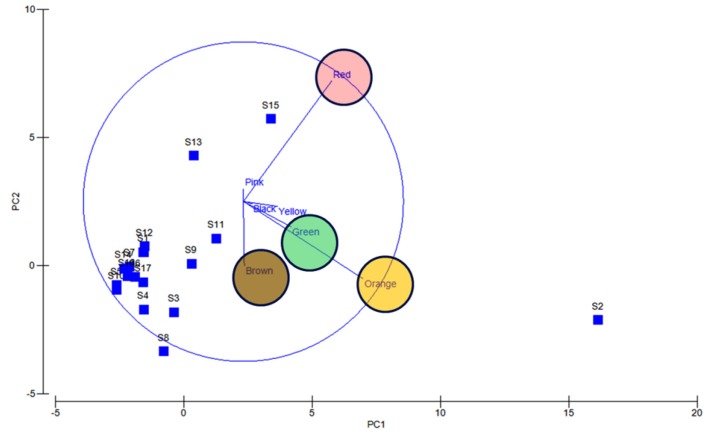
PCA analysis reveals orange, red, green and brown colored bacterial dominance. Blue lines and circles indicate the dominance and distribution patterns of pigment bacteria at different stations.

Apparently, most of the isolates found to be uncultivable upon first streaking, and some after longer period or even on first isolation as is the frequently observed case with red pigment isolates (Supplementary Figures S4 and S5). Also, several potential agar degrading bacteria were observed along with the pigmented bacterial isolates, hence sometimes the plates become liquid (Supplementary Figure S6). Principal component analysis also indicated the dominance of orange, red, green, and brown pigmented bacterial isolates in South Andaman Island (Figure 6).

**Figure 7:**
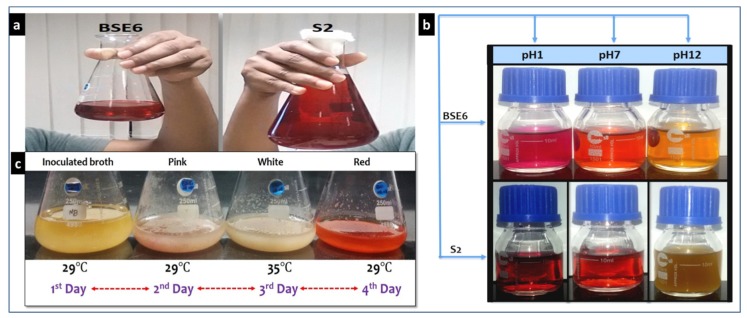
Red pigment extracts of strains BSE6 & S2.1 (**a**), and their pigment color variation at different pH’s (**b**); Heat stimulated pigmentation in strain BSE6.1 (**c**).

Strain BSE6.1 showed temperature induced red pigment (Figure 7a,c). Strain S2.1 displayed pink colour in nutrient broth supplemented with calcium (CaCO_3_). Both these strains showed a dark red pigment at pH 1 and yellow pigmentation at pH 12 (Figure 7b). Newly formed colonies of strain S2.1 shows a pink colour, then becomes blood red, and later brownish red after few months. Surfaces of this strain colonies also display metallic greenish and golden yellow sheen coating. Whereas, strain BSE6.1 produces pink color in Minimal broth with 2% NaCl, and red pigment production in all other media. It also produces tiny white colored spores on both broth and agar media after 7 or 10 days of incubation.

### 4.2. Antibacterial Activity of the Pigment

**Figure 8:**
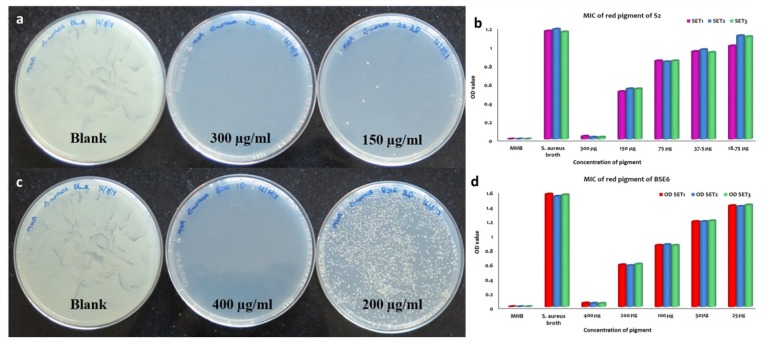
MBC and MIC of S2.1 extracts against *S. aureus* (**a**,**b**). MBC and MIC of BSE6.1 extracts against *S. aureus* (**c**,**d**).

Out of fourteen pigment bacterial strains tested, two red pigment strains displayed effective antibacterial application against all the tested pathogens, including *Enterococcus faecalis* MTCC9845, *Escherichia coli* MTCC730, *Klebsiella pneumoniae* subsp. *pneumoniae* MTCC109, *Salmonella enterica* MTCC1165, *Salmonella enterica* ser. *typhi* MTCC733, *Salmonella enterica typhimurium* MTCC98, *Staphylococcus aureus* subsp. *aureus* MTCC1430 and *Streptococcus mutans* MTCC890. MIC and MBC analysis of both pigment extracts obtained from strain S2.1 and BSE6.1 displayed complete inhibition of *S. aureus* at the ranges between 150 to 400 µg/mL concentrations, respectively (Figure 8). Prodigiosin extracts (with concentrations of 50 to 400 µg/mL) obtained from various marine bacteria are reportedly displayed effective antibacterial against different pathogenic bacteria [5]. The inhibition zones (20 to 28 mm) observed in this study are greater than the previous studies [5,24]. Thus, strains S2.1 and BSE6.1 are offering great demand for drug development.

### 4.3. Application of Pigments as Strainers and Food Colorants

**Figure 9:**
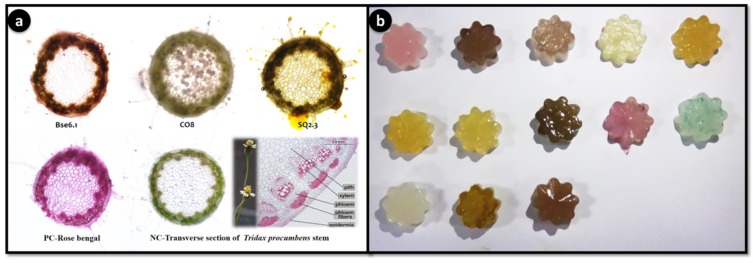
Staining application of potential pigment strains. Transverse stions of *Tridax procumbens* stem. PC: positive control-Rose bengal; NC: negative control-*Tridax* stem (**a**); Food colorant application as agar jellies prepared using extractions of different pigment bacterial strains (pink color jellies represent strain BSE6.1. & S2.1) (**b**).

The application of food colorant and staining properties of different pigmented bacterial extracts showed potential food colorant and staining properties (Figure 9). For the first time, this study demonstrated the natural staining of stem stions of *Tridax procumbens.* Agar jellies colored with pigments have lasted up to 3 months with intact pigment, indicating that these pigments can be used as food grade pigments. Staining of *T. procumbens* stem also displayed three strains BSE6.1, CO8 and SQ2.3 have strong ability to stain epidermis and parenchyma cells. Therefore, these pigments can be used for staining applications in laboratories and education centres.

### 4.4. Purification and Identification of the Pigment

Both strains were initially cross streaked to investigate the antagonism between each other. However, antagonism was not observed between these two strains (Figure 4a). Crude pigments were purified on TLC and observed two distinct red pigment bands with R*f* values of 0.6 for both strains (Figure 4b). UV-Visible spectroscopic analysis with purified pigment compounds of BSE6.1 and S2.1 revealed maximum absorption spectra at 528 nm and 538 nm, respectively (Figure 4c,d). Several researchers reported that prodigiosin is a primary pigment in *Serratia* sp. and has the maximum absorbance at 535 nm [25].

Further analysis with HP-LC, GC-MS, FT-IR and proton NMR revealed highly similar chemical properties among these two strains. The major red pigmented compound constituents were identified by HPLC at the indicated retention times of 8.09 min (m/z 324, prodigiosin) for strain BSE6.1 and 9.72 min (m/z 338, 2-methyl-3-hexyl prodiginine) for strain S2.1. These particular retention peaks were separated and FT-IR, NMR analysis were conducted. Major chemical compounds responsible for red pigment production in both red pigmented strains BSE6.1 and S2.1 were also identified as prodiginine constituents in GC-MS. Mass spectrometry analysis revealed presence of 2-methyl-3-propyl prodiginine compound (m/z 296) at 6.36 RT and 24.50 RT in strain S2.1 and in strain BSE6.1, streptophenazine, a yellow compound (m/z 496) and 2-methyl-3-butyl prodiginine or 2-methyl-3-propyl prodiginine, a prodiginine components of red pigment compound (m/z 310 or m/z 296) were detected at 35.27 RT and 17.18 or 18.61 respectively. These spectra are similar to the published prodiginine constituents [24]. FTIR and NMR results are also in accordance with GC-MS library with slight variation.

The broad stretching IR peaks similar to prodigiosin compound were detected at frequency range 3386–644 cm^−1^ in both the red pigment extracts of S2.1 and BSE6.1. Weak absorption at 2500–1700 cm^−1^ was detected in both bacterial strains. The prodiginine compound spectra observed for strain BSE6.1 was 3386, 2975, 1647, and 1087, and for BSE6.1, 3413, 2976, 1646, and 1086 (Figure 5a,b). These broad IR peaks are in accordance to the prodigiosin peaks 3105, 2928, 1644, 1602 and 1125 reported in a previous study [26]. Song et al. (2006) reported that *Serratia* sp. KH95 was dominated by strong bands at 2928 cm^−1^ (aromatic CH) and 1602 cm^−1^ (aromatic C = C) [27]. The pigment was identified to be prodigiosin on the basis of spectroscopic data of ^1^H-NMR (400 MHz, CDCl_3_, δ). The ^1^H-NMR spectra of red pigment extracted from *Zooshikella* sp. strain S2.1 and *Streptomyces* sp. strain BSE6.1 are comprised of almost similar functional groups and characteristic chemical shifts (ppm) with consistent functional groups. Two major spectral signals were detected at ppm 3.430 and between 3.091 to 3.252 (Figure 5c,d); confirming the presence of red pigmented prodiginine chemical components as reported earlier [26]. Prodigiosin compounds give a typical signals of NMR protons on the three heterocyclic structures between 6–7.3 ppm for *Z. ganghwensis*, *Pseudoalteromonas* sp., and *Pseudoalteromonas rubra* [26,28,29]. Song et al. (2006) reported that, a chemical shift of the methoxy group in prodigiosin exhibited peak at 4.04 ppm as singlet in *Serratia* sp. KH-95 [27]. However, slight chemical shifts observed in the pigment compound of both strains S2.1 and BSE6.1 indicating a different prodigiosin chemical component.

### 4.5. Identification of the Potent Strains S2.1 and BSE6.1

**Figure 10:**
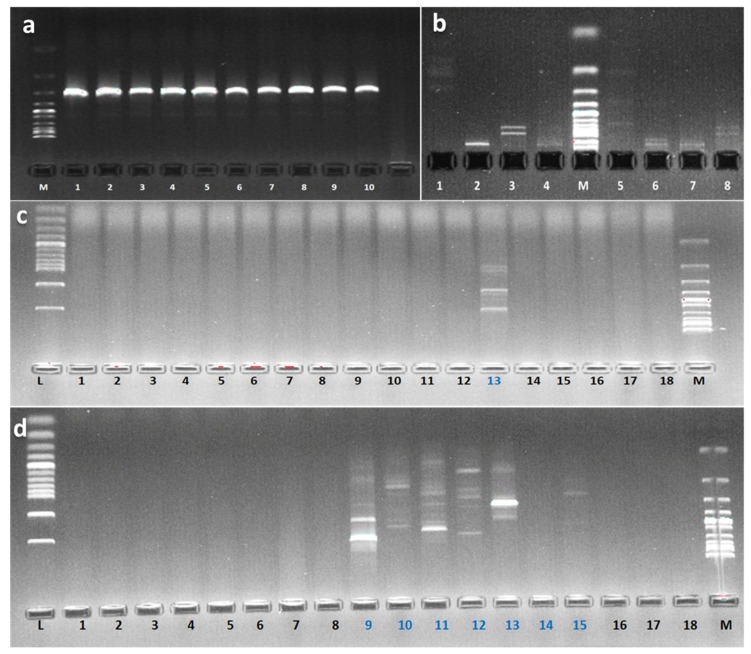
PCR amplified products of 16S rRNA. M: Ladder 1 Kb; Numbers: 1, (**a**); RFLP banding patterns of S2.1 (1 to 4) and BSE6.1 (5 to 8) digested with endonucleases *AluI*, *HindIII*, *BamHI* and *EcoRI*. M: Ladder 100 bp (**b**); Agarose gel electrophoresis of amplified DNA products of BSE6.1 1 digested with different RAPD primers. L: Ladder 100 bp & M: Ladder 1 Kb (**c**); Agarose gel electrophoresis of amplified DNA products of S2.1 digested with different RAPD primers. L: Ladder 100 bp & M: Ladder 1 Kb (**d**).

PCR amplification was successful and amplified a 1500 bp size of 16S rRNA gene (Figure 10a). Selected pigmented bacterial strains were identified up to genus or species level (Table 1). Sequence analysis of the 16S rRNA gene revealed that strain S2.1 and BSE6.1 represents a novel lineage within the family Hahellaceae and Streptomycetaceae respectively. Strain S2.1 was Gram-negative, intensive blood red pigmented, that showed the sequence similarity of 99.77% with the closely related species of *Zooshikella marina* strain JC333^T^.

RAPD investigation revealed that out of twenty tested RAPD primers, OPA-07 GAAACGGGTG, OPA-12 TCGGCGATAG, OPB-01 GTTTCGCTCC, OPB-15 GGAGGGTGTT, OPC-05 GATGACCGCC, OPD-10 GGTCTACACC and OPE-11 GAGTCTCAGG have generated specific banding patterns for BSE6 & S2 bacterial strains (Figure 10c,d). Similarly, restriction enzymes *AluI*, *HindIII*, *BamHI* and *EcoRI* also resulted the different banding pattern confirming that these two bacterial strains are distinct each other (Figure 10b).

The first description of genus *Zooshikella* was reported in 2003 as *Zooshikella ganghwensis* gen. nov., sp. nov., under the class gamma-Proteobacteria [30]. Type strains of *Z. ganghwensis* were first isolated from coastal sediment sample collected from the getbol of Ganghwa Island in Korea [30]. Phylogenetically, *Zooshikella* shows a close proximity and lineage with red pigmented bacterial genus *Hahella*. So far only three species, *Z. ganghwensis* JC2044^T^ [30], *Z. marina* JC333^T^ [31], and *Z. rubidus* S1-1 [24] have been reported under the genus *Zooshikella*; while taxonomic status of the latter species name is yet to be confirmed by polyphasic taxonomic approach. All the three species of this genus *Zooshikella* were isolated from coastal sediment samples alone. The genus *Zooshikella* has not been explored much, making it an unexplored source of pharmaceutically important pigment compounds and food grade colorants. The major chemical constituents reported in the *Zooshikella* species were prodigiosin and cycloprodigiosin. These compounds have displayed potential biological activities such as anti-oncogenic activity against human melanoma cells [24] and other human cancer cell lines, SK-BR-3, HCT-116, SK-OV-3, HeLa, HepG2, and SK-MEL-5 [26], antibacterial activity against several microbial species [24,32,33], and dyeing properties [34]. Comparative to *Zooshikella* type strain JC2044, strain S2.1 is able to tolerate maximum temperature of 35 °C and displayed antibacterial inhibition activity range of 20–28 mm against investigated pathogens. The two strains, S2.1 and BSE6.1 producing prodigiosin-like pigments are very close to already known strains on the basis of 16S rRNA gene sequences and the observed antibacterial effect of prodigiosin pigment has been also reported in previous studies.

Previous study reported that the majority of pigmentation was remained in the microbe-rich mucus pellet of rays [35]. However, we have not found such an incident for fish samples, but it was evident that the pigmented bacterial population was greater in sediment, invertebrates and seawater samples. The inhibited growth of bacteria on media containing seawater indicates that they might be of terrestrial origin [36], and this incident was not observed for any of pigmented bacterial isolates in this study. Antagonistic effects on indigenous pigment bacterial isolates were observed in a previous study [37], however, in the present study such case was not observed. The RAPD primers OPA-07 GAAACGGGTG, OPA-12 TCGGCGATAG, OPB-01 GTTTCGCTCC, OPB-15 GGAGGGTGTT, OPC-05 GATGACCGCC, OPD-10 GGTCTACACC and OPE-11 GAGTCTCAGG and restriction enzymes *AluI*, *HindIII*, *BamHI* and *EcoRI* could be used for effective differentiation of members of *Zooshikella* and *Streptomyces* species in Andaman waters.

Whole genome sequencing of strain S2.1 using Illumina platform revealed absence of 365 genes and significantly 1989 genes were not detected in KEGG pathway database (Ramesh et al., 2019c). Thus, strain S2.1 was proposed as a novel species as *Z. andamani* which is also yet to be verified by polyphasic taxonomical approach. While, strain BSE6.1is not similar to prodigiosin producing *Streptomyces griseoviridis*, thus whole genome of BSE6.1 is under process to confirm the novelty level of this strain. Conversely, the pigments of strain S2.1 and BSE6.1 can be used as food colorants and as natural stains in laboratorial purposes. Further, *Zooshikella* sp. strain S2.1 and *Streptomyces* sp. strain BSE6.1 could be used for genetic engineering studies to improve pigment production for various applications.

## 5. Conclusions

The present project work report reveals the dominant distribution of pigment bacteria in sediment and invertebrate samples collected from different locations in South Andaman. Among different pigment types, red, orange, brown and yellow were predominate pigment type bacterial isolates were isolated. Among 17 study locations, pigment bacteria were higher and varied pigment types were observed in Burmanallah station. Two red pigment producing strains S2.1 and BSE6.1 have displayed promising multifaceted applications. Thus, these two strains are selected for further applications. In due course of time, these strains will be deposited in Microbial Type Culture Collection, India for the benefit of researchers and industries.

The advanced biotechnological techniques would open new avenues for large scale industrial production of natural pigments for various applications and to substitute the synthetic pigments. This study provides a detailed data on genetic diversity of several potential marine pigmented bacterial species like *Zooshikella* and *Streptomyces*, and possibility of isolation of some novel species from diverse marine samples of Andaman Islands. The isolated cultures are maintained as a culture collection for future applications and reference. Based on the baseline investigations, it is evident that pristine Andaman Islands offer a wide variety of pigmented bacteria with potential biomedical applications. Thus, research on this aspect is needed to be extended to explore novel pigment bacteria which will offer natural pigments for food and drug applications.

## Figures and Tables

**Table 1:** Identification of pigmented bacterial strains using 16s rDNA sequence.

S.No	Pigmented Bacterial Strain	Isolation Source	GenBank Accession Number	Sequence Similarity %	Identified as
1.	SW1	Seawater	MK680109	99.62	*Pseudoalteromonas peptidolytica*
2.	SW2	Seawater	MK680110	99.02	*Pseudoalteromonas peptidolytica*
3.	SW4	Seawater	MK680111	99.27	*Gordonia terrae*
4.	CO2	*Conus miles*	ND		ND
5.	SQ2.3	Juvenile squid	ND		ND
6.	PC3		MK680107	96.66	*Nitratireductor basaltis*
7.	NBE2	Unidentified nudibranch egg mass	MK680106	98.71	*Salinicoccus roseus*
8.	MSW2	Seawater	MK680105	99.42	*Idiomarina sediminum*
9.	CSW3	Seawater	MK680104	99.34	*Idiomarina sediminum*
10.	BSP1	Unidentified violet Sponge	MK680103	99.41	*Pseudomonas aeruginosa*
11.	CO8	*Conus miles*	ND		ND
12.	DA1	Seawater	ND		ND
13.	BSE6.1	Sediment	MK951781	99.71	*Streptomyces* sp.
14.	S2.1	Sediment	MK680108	99.77	*Zooshikella marina*
15.	BSC	*Holothuria artra*	MK680112	96.64	*Serratia marcescens*
16.	AB4	*Acetabularia acetabulum*	MK680101	95.65	*Photobacterium ganghwense*
17.	AB5	*Acetabularia acetabulum*	MK680102	97.87	*Photobacterium ganghwense*

## Supplementary Materials

The following are available online at https://www.mdpi.com/2076-2607/8/4/556/s1.
