# Supplementary material for: Applications of Prodigiosin Extracted from Marine Red Pigmented Bacteria Zooshikella sp. and Actinomycete Streptomyces sp
Source: Microorganisms. 2020 Apr 13;8(4):556. doi: 10.3390/microorganisms8040556 (PMC7232315; doi:10.3390/microorganisms8040556)
Supplement: Supplementary file 1 [file microorganisms-08-00556-s001.pdf]

Table S1. Different biotic and abiotic samples tested for pigmented bacterial isolation.

S. No	Sample name	Sample code	Sampling station
Floral samples			
1.	Green algae *Enteromorpha compressa*	JGA	Junglighat
2.	Cyanobacterial mat	BCM	Burmanallah
3.	Perfume seaweed	BPS	Burmanallah
4.	Mangrove *Rhizophora apiculata*	BRA	Burmanallah
5.	Seaweed *Acetabularia* sp.	AB	Burmanallah
6.	*Symploca hydnoides* (non-heterocystous cyanobacterium)	BS	Burmanallah
7.	Bifurcated seaweed *Dictyopteris acrostichoides*	BFS	Wandoor
8.	*Halimeda tuna* green algae	TU	Wandoor
9.	*Padina tetrastromatica* seaweed	PA	Wandoor
Faunal samples			
10.	*Uca* sp.	CS	Mini Bay
11.	Nudibranch	NB	Burmanallah
12.	Ascidian	AC	Burmanallah
13.	Green algae *Acetabularia calyculus*	AS	Burmanallah
14.	*Thallasisa* seagrass	ST	Burmanallah
15.	Pinctada margaritifera (black-lip pearl oyster)	PB	Burmanallah
16.	Seawater	JW	Junglighat
17.	*Arothron immaculatus* juvenile (puffer fish)	AH	Junglighat
18.	Squid Juvenile-big	SQ1	Junglighat
19.	Squid Juvenile-small	SQ2	Junglighat
20.	*Uca* sp. (fiddler crab)	PU	Pongi Balu
21.	Purple climber crab (*Metopograpsus messor*)	PVC	Pongi Balu
22.	*Conus araneosus*	PBC	Pongi Balu
23.	*Pteraeolidia ianthina* Blue dragon slug	PNB	Pongi Balu
24.	Soft coral unidentified	SCU	Burmanallah
25.	Brain coral	BBC	Burmanallah
26.	Soft coral cup shaped	SCC	Burmanallah
27.	*Pseudosquilla ciliata* (Green mantis shrimp)	MS	Wandoor
28.	Rui fish Gill	RFG	Burmanallah
29.	Blue sponge unidentified	BSN	North Bay
30.	Orange sponge unidentified	OSN	North Bay
31.	Algal Bloom (*Protoperidinium* sp.)	JAB	Junglighat
32.	Algal Bloom (*Akashia-Gymnodinium sanguineum*)	MAB	Mini Bay
33.	Mixed algal culture (*Nitschia*)	MAC	Mini Bay
34.	Sponge (Oyster tank)	SP1	Mini Bay

35.	Lugworm (*Arenicola marina*)	LBW	Loha Behara
36.	*Ligia dentipes* (Sea Slater)	LE	Wandoor
37.	Soldier Crab (*Dotilla myctiroides*)	LBC	Loha Behara
38.	Heart urchin *Maretia planulata*	LBS	Loha Behara
39.	Nudibranch *Polybranchus orientalis* egg case	BNE	Burmanallah
40.	*Gymnodoris subflava* sea slug	BNB	Burmanallah
41.	*Leiognathus* sp. Pony fish	MLF	Mini Bay
42.	Sponge violet color	BSP1	Burmanallah
43.	Sponge (coral type)	BSP2	Burmanallah
44.	Seacucumber Black red	SC1	Wandoor
45.	Stichopus vastus (Curryfish- sea cucumber)	SC2	Wandoor
46.	Seacucmber yellow brown dots	SC3	Wandoor
47.	Dead *Echinothrix calamaris* (banded sea urchin)	KSU	Kodiyaghat
48.	Polychaete worms	PW	Kodiyaghat
49.	*Aurelia aurita* (Moon jelly)	CJP	Chidiyatapu
50.	Sponge1	SP1	Burmanallah
51.	Sponge2	SP2	Burmanallah
52.	Sponge3	SP3	Burmanallah
53.	Sponge	SP4	Burmanallah
54.	Sponge	SC4	Burmanallah
55.	Seacucumber	SSB	Burmanallah
56.	*Linckia laevigata* (Blue Sea Star)	SSRS	Burmanallah
57.	Polychaete	PC	Burmanallah
58.	*Conus miles*	CO	Burmanallah
59.	Crab	CR	Burmanallah
60.	Yellow sponge *Stylissa massa*	KDYSP	Kurma Dera Beach
61.	Dead coral	KDDC	Kurma Dera Beach
62.	Black sponge1 (unidentified)	BSP	Burmanallah
63.	Sea cucumber *Holothuria artra*	BSC	Burmanallah
64.	Black sponge2 (unidentified)	BSPB	Burmanallah
65.	Rock oyster *Saccostrea cucullata*	BRO	Burmanallah
66.	Coral *Porites* sp.	BCP	Burmanallah
67.	Brittle star *Ophiocoma dentata*	BOS	Burmanallah
68.	Cement colour sponge	BCSP	Burmanallah
69.	Nudibranch egg mass (Unidentified)	BUE	Burmanallah
70.	*Obelia* sp. (red extract in sea water)	NBO1	North Bay
71.	*Obelia* sp. (only *Obelia* extract)	NBO2	North Bay
72.	Unidentified nudibranch egg mass	NBE	North Bay
73.	Polychaete worms on PVC tubes	NBP	North Bay
74.	Comb Jellies	NBCJ	North Bay
75.	*Halichoeres* sp. (Wrasse fish)	MBW	Mini Bay
76.	*Conus miles*	CCO	Corbyn's Cove

77.	Edible red jellyfish *Crambione mastigophora*	PRJ	Pongi Balu
Abiotic samples			
78.	Muscovite (common mica)	CHWR	Chouldari
79.	Seawater	DSW	Dignabad
80.	Sediment	DSE	Dignabad
81.	Seawater	MSW1	Marina Park
82.	Sediment 1	MSE1	Marina Park
83.	Seawater	MSW2	Marina Park
84.	Sediment 2	MSE2	Marina Park
85.	Seawater	MSW3	Marina Park
86.	Sediment 3	MSE3	Marina Park
87.	Seawater	SSW	Science Center
88.	Sediment	SSE	Science Center
89.	Sediment	CSE	Corbyn's Cove
90.	Seawater	CW	Corbyn's Cove
91.	Sediment 1	KSE1	Kodiyaghat
92.	Sediment 2	KSE2	Kodiyaghat
93.	Sediment 3	KSE3	Kodiyaghat
94.	Sediment 4	KSE4	Kodiyaghat
95.	Sediment 5	KSE5	Kodiyaghat
96.	Sediment 6	KSE6	Kodiyaghat
97.	Sediment 7	KSE7	Kodiyaghat
98.	Seawater 1	KSW1	Kodiyaghat
99.	Seawater-decomposed smell	KSW2	Kodiyaghat
100.	Sediment	CSE1	Chidiyatapu
101.	Sediment	CSE2	Chidiyatapu
102.	Sediment	CSE3	Chidiyatapu
103.	Sediment	CSE4	Chidiyatapu
104.	Sediment	CSE5	Chidiyatapu
105.	Sediment	CSE6	Chidiyatapu
106.	Sediment	CSE7	Chidiyatapu
107.	Seawater	CSW1	Chidiyatapu
108.	Seawater	CSW2	Chidiyatapu
109.	Seawater	CSW3	Chidiyatapu
110.	Sediment	CHSE1	Chouldari
111.	Sediment	CHSE2	Chouldari
112.	Seawater	CHSW1	Chouldari
113.	Seawater	CHSW2	Chouldari
114.	Sediment	KDSE1	Kurma Dera Beach
115.	Sediment	KDSE2	Kurma Dera Beach
116.	Sediment	KDSE3	Kurma Dera Beach
117.	Seawater	KDSW1	Kurma Dera Beach
118.	Yellow seawater	KDYSW1	Kurma Dera Beach
119.	Dark yellow seawater	KDYSW2	Kurma Dera Beach

120.	Oily yellow seawater	KDOYSW	Kurma Dera Beach
121.	Sediment 1	GSE1	Guptapara
122.	Sediment 2	GSE2	Guptapara
123.	Sediment 3	GSE3	Guptapara
124.	Seawater	GSW	Guptapara
125.	Seawater 1	MSMW1	Manjery
126.	Sediment 1	MSE1	Manjery
127.	Sediment 2	MSE2	Manjery
128.	Seawater 2	MSMW2	Manjery
129.	Sediment 3	MSE3	Manjery
130.	Seawater 1	PSW1	Pongi Balu
131.	Seawater 2	PSW2	Pongi Balu
132.	Sediment	PSE	Pongi Balu
133.	Seawater 1 "0" meter	JSW1	Junglighat
134.	Seawater 2 "6" meter	JSW2	Junglighat
135.	Sediment "7" meter	JSE	Junglighat
136.	Sediment	BS1	Burmanallah
137.	Sediment	BS2	Burmanallah
138.	Sediment	BS3	Burmanallah
139.	Sediment	BS4	Burmanallah
140.	Sediment	BS5	Burmanallah
141.	Sediment	BS6	Burmanallah
142.	Sediment	BS7	Burmanallah
143.	Sediment	BS8	Burmanallah
144.	Seawater	BSW	Burmanallah
145.	Seawater	BSW1	Burmanallah
146.	Sediment	BSE1	Burmanallah
147.	Seawater	BSW2	Burmanallah
148.	Sediment	BSE2	Burmanallah
149.	Seawater	BSW3	Burmanallah
150.	Sediment	BSE3	Burmanallah
151.	Seawater	BSW4	Burmanallah
152.	Sediment	BSE4	Burmanallah
153.	Seawater	BSW5	Burmanallah
154.	Sediment	BSE5	Burmanallah
155.	Seawater	BSW6	Burmanallah
156.	Sediment	BSE6	Burmanallah
157.	Sediment 1	S1	Burmanallah
158.	Sediment 2	S2	Burmanallah
159.	Sediment 3	S3	Burmanallah
160.	Sediment 4	S4	Burmanallah
161.	Seawater	SW	Burmanallah
162.	Sediment	S1	Burmanallah
163.	Sediment	S2	Burmanallah
164.	Sediment	S3	Burmanallah

165.	Sediment	S4	Burmanallah
166.	Sediment	S5	Burmanallah
167.	Sediment	S6	Burmanallah
168.	Sediment	S7	Burmanallah
169.	Sediment	S8	Burmanallah
170.	Sediment	S9	Burmanallah
171.	Sediment	S10	Burmanallah
172.	Sediment	S11	Burmanallah
173.	Sediment	S12	Burmanallah
174.	Sediment	S13	Burmanallah
175.	Sediment	S14	Burmanallah
176.	Sediment 1	SE1	Wandoor
177.	Sediment 2	SE2	Wandoor
178.	Seawater	LBSW	Loha Behara
179.	Yellow seawater	LBYSM	Loha Behara
180.	Sediment	BSE	Loha Behara

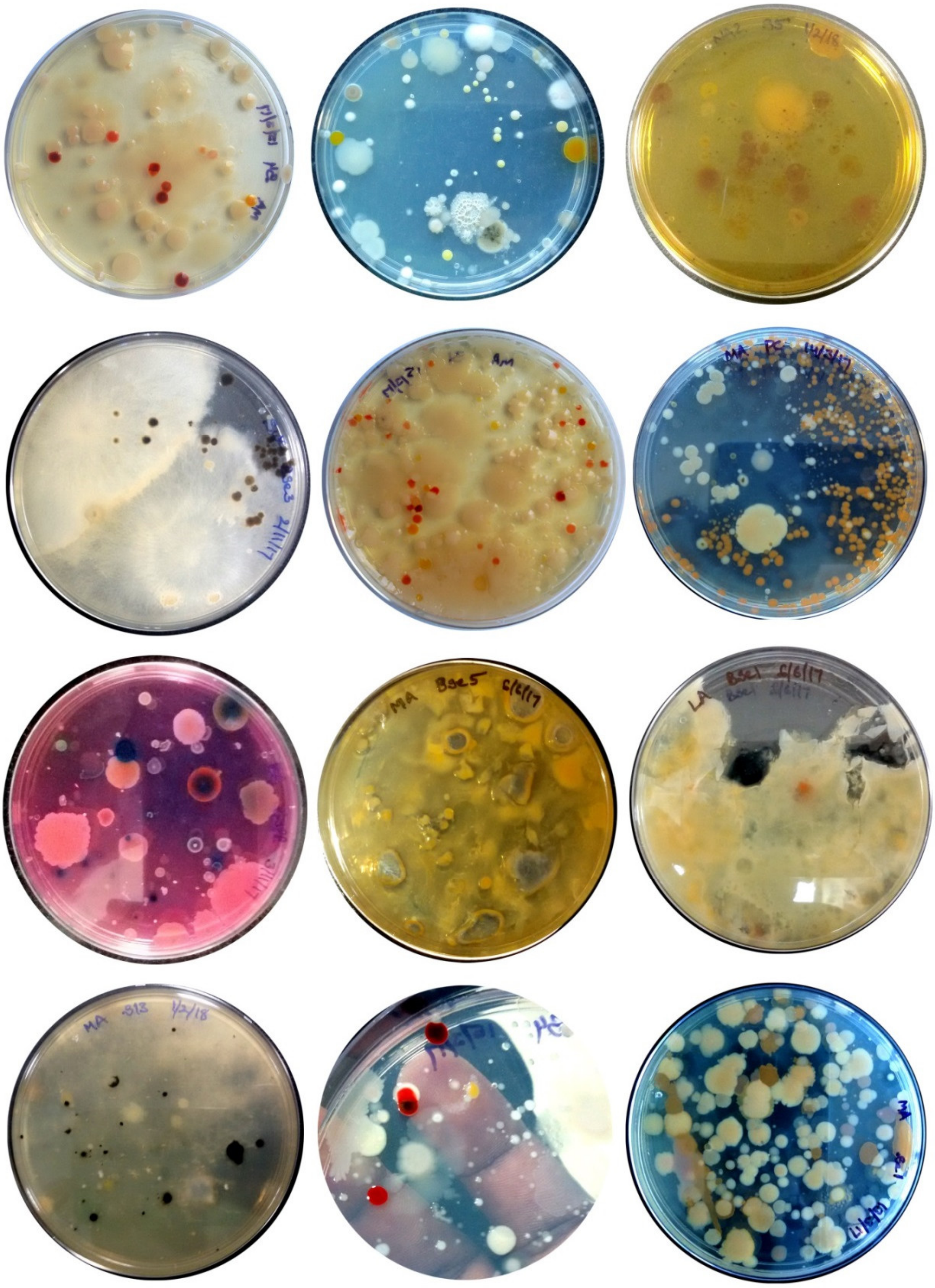

Figure S1. Red, orange and black pigments producing marine strains.

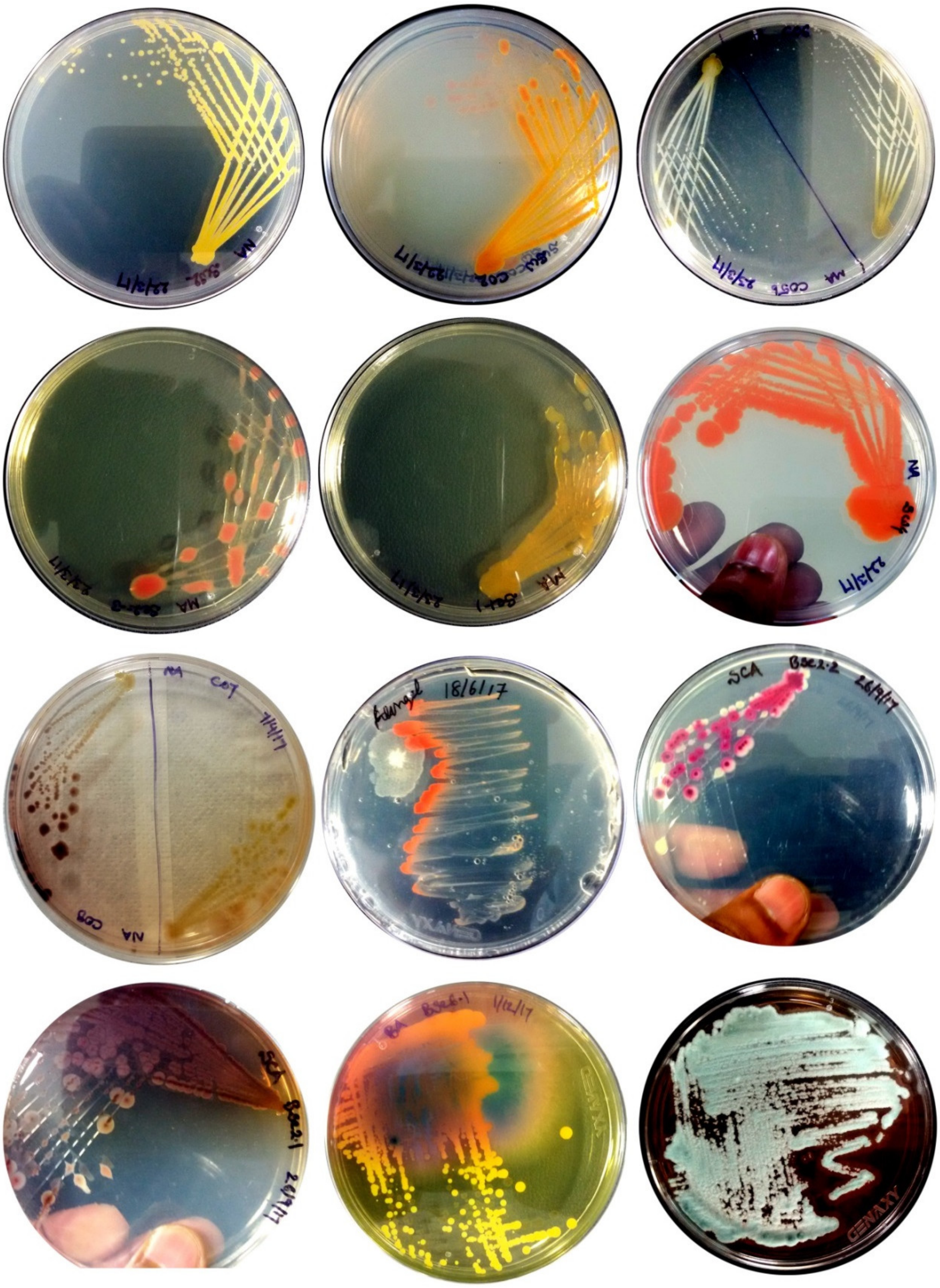

Figure S2. Yellow, orange, violet, pink red, pale blue pigments producing marine strains.

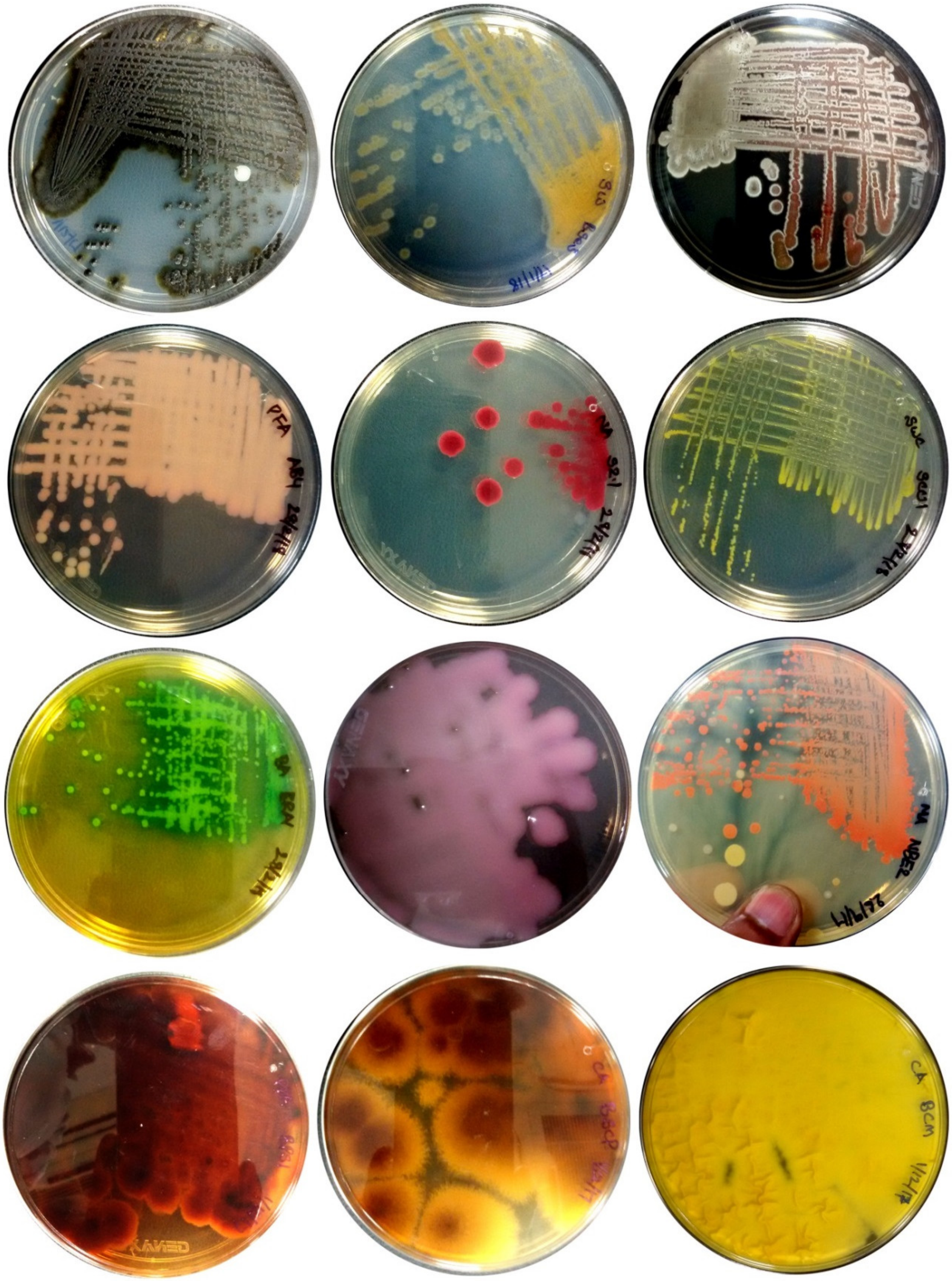

Figure S3. Black, pink, red, green and dark brown pigments producing marine strains.

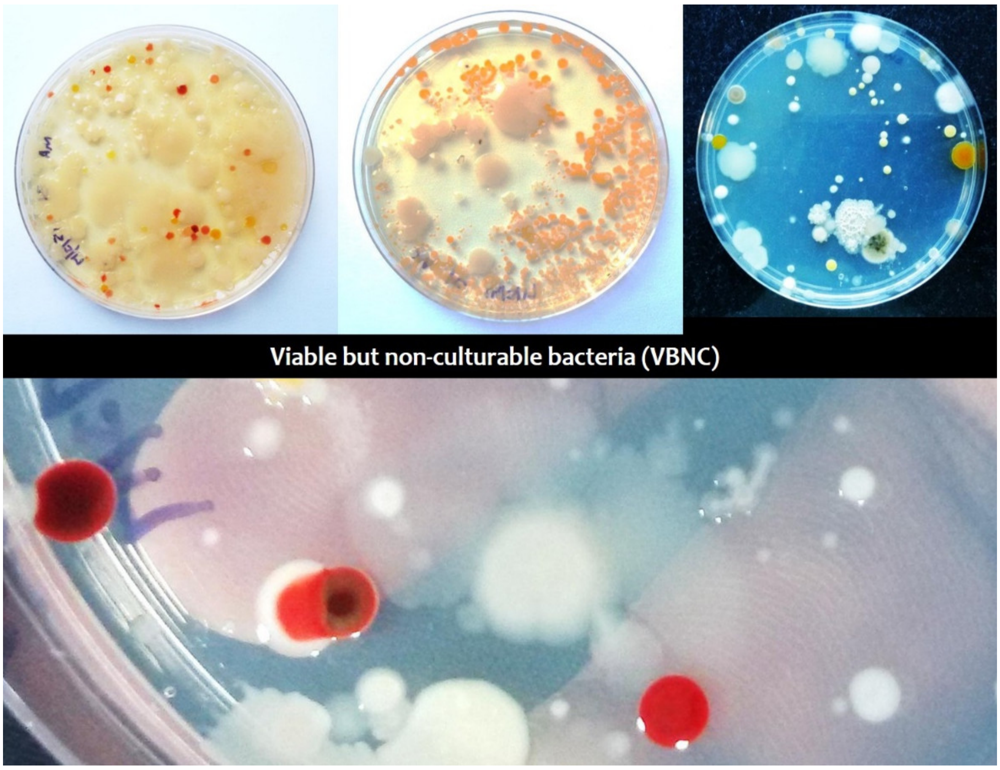

Figure S4. Viable but non-culturable orange and red pigment bacterial isolates.

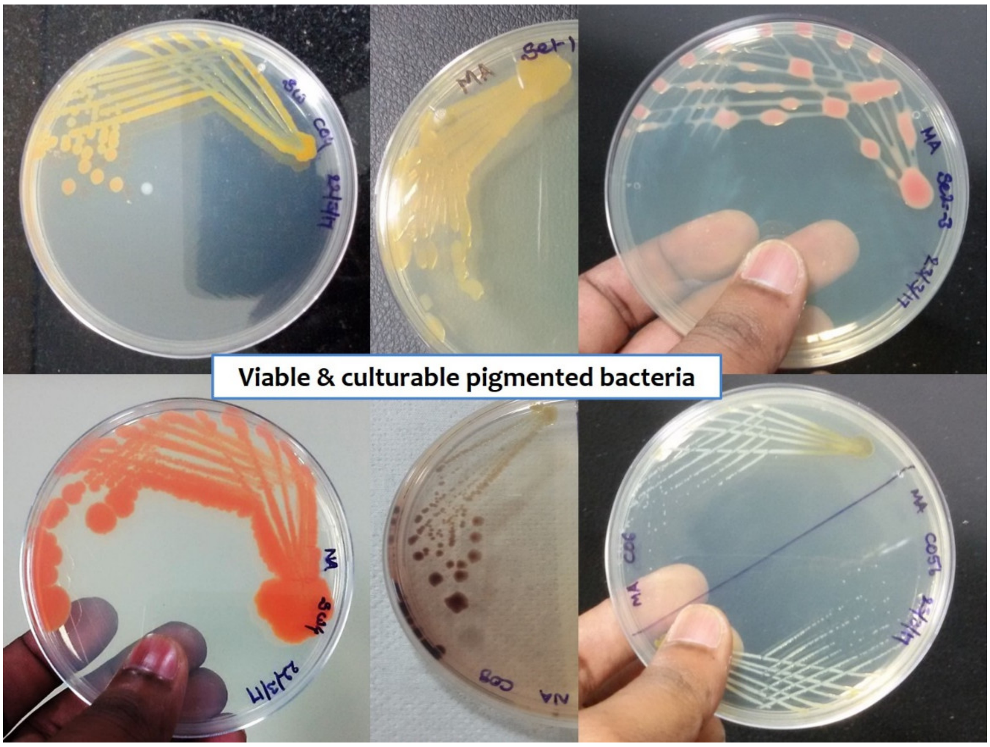

Figure S5. Some viable and culturable pigmented bacterial isolates.

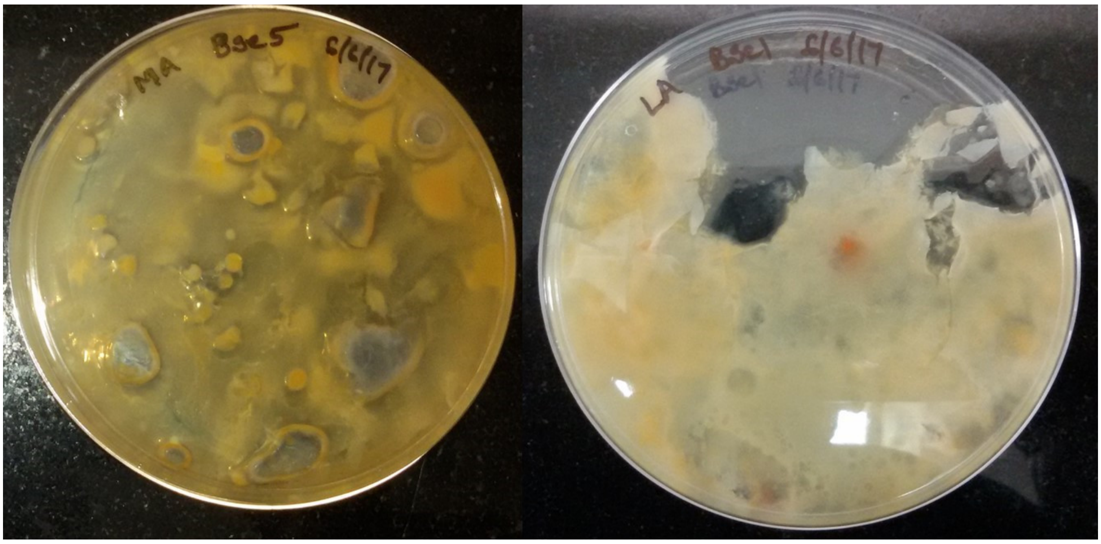

Figure S6. Potential agar degrading bacteria.
